# Solid state NMR of isotope labelled murine fur: a powerful tool to study atomic level keratin structure and treatment effects

**DOI:** 10.1007/s10858-016-0056-7

**Published:** 2016-10-03

**Authors:** Wai Ching Veronica Wong, Aurimas Narkevicius, Wing Ying Chow, David G. Reid, Rakesh Rajan, Roger A. Brooks, Maggie Green, Melinda J. Duer

**Affiliations:** 1 Department of Chemistry, University of Cambridge, Lensfield Road, Cambridge, CB2 1EW UK; 2 Central Biomedical Resources, School of Clinical Medicine, University of Cambridge, West Forvie Building, Forvie Site, Robinson Way, Cambridge, CB2 0SZ UK; 3 Department of Trauma and Orthopaedic Surgery, Addenbrooke’s Hospital, University of Cambridge, Cambridge, CB2 0QQ UK

**Keywords:** Carbon-13, Cosmetics, Fur, Hair, In vivo, Keratin, Labelling, Mouse

## Abstract

We have prepared mouse fur extensively ^13^C,^15^N-labelled in all amino acid types enabling application of 2D solid state NMR techniques which establish covalent and spatial proximities within, and in favorable cases between, residues. ^13^C double quantum–single quantum correlation and proton driven spin diffusion techniques are particularly useful for resolving certain amino acid types. Unlike 1D experiments on isotopically normal material, the 2D methods allow the chemical shifts of entire spin systems of numerous residue types to be determined, particularly those with one or more distinctively shifted atoms such as Gly, Ser, Thr, Tyr, Phe, Val, Leu, Ile and Pro. Also the partial resolution of the amide signals into two signal envelopes comprising of α-helical, and β-sheet/random coil components, enables resolution of otherwise overlapped α-carbon signals into two distinct cross peak families corresponding to these respective secondary structural regions. The increase in resolution conferred by extensive labelling offers new opportunities to study the chemical fate and structural environments of specific atom and amino acid types under the influence of commercial processes, and therapeutic or cosmetic treatments.

**Electronic supplementary material:**

The online version of this article (doi:10.1007/s10858-016-0056-7) contains supplementary material, which is available to authorized users.

## Introduction

Keratinous tissues, including skin, hair, fur, nails, claws, hooves, and feathers, play essential and varied biological roles as connective, structural, protective, and insulating materials. They are important as sources of raw materials, in medical and veterinary therapy, as well as in cosmetic industry and science. Relationships between keratin chemical composition (Robbins 2012), molecular structure and supramolecular organization (Bragulla and Homberger 2009; Popescu and Hocker 2007) and material properties (McKittrick et al. 2012), genomics (Schweizer et al. 2006), and the molecular basis of cosmetic treatments (Miranda-Vilela et al. 2014), have all been extensively reviewed. The most important component of the softer keratins such as hair is α-keratin, comprising of three-domains: the central α-helical rod domain, and N- and C-terminal “head” and “tail” domains. A pair of individual polypeptide chains, one acidic (Type I) and one neutral or basic (Type II), in the rod domain dimerize to form “coiled coils” which self-assemble into protofilaments. Protofilaments assemble, laterally and end-to-end, to form intermediate filaments (IFs), the fundamental structural unit of keratin fibres. IFs are embedded in a matrix containing proteins with high levels of glycine and tyrosine, and cysteine, termed keratin associated proteins (KAPs). In hard tissues, the head and tail domains of the keratin proteins crosslink with the matrix.

Solid state NMR (ssNMR) has been applied to characterize, compare, and tentatively assign spectra from hair, hoof, and feathers. The secondary structure content is predicted to be highly heterogeneous, with α-helical rod domains co-existing with β-sheet, random coil, and turn, structures in the keratin head and tail domains and in the matrix proteins. This heterogeneity is reflected in the partial resolution of the amide carbon signal into higher frequency (α-helix) and lower frequency (β-sheet/random coil) components (Kricheldorf and Muller 1984). Different chemically fractionated wool keratin components display very different overall secondary structural ratios. The reduced, S-carboxymethyl protected low sulfur (SCMKA corresponding to the hard keratin of the IF), and high sulfur (SCMKB) fractions are predominantly α-helical, while the Gly, Tyr-rich, fractions are predominantly β-sheet/disordered (Yoshimizu and Ando 1990; Yoshimizu et al. 1991b). Processes which model cosmetic treatments, such as fibre stretching (Yoshimizu et al. 1991a), and reduction–oxidation “permanent waving” (Nishikawa et al. 1998a) of hair, are reported to increase β-sheet/disorder content. The low sulfur and high sulfur fractions of human hair intermediate filaments can be solubilized; solution state ^13^C NMR suggests that the former is ca. 40 % α-helical, the latter random coil (Nishikawa et al. 1998b). In solubilized α-helical coiled coil fractions no signals are detectable from amino acids in the rigid, structured, rod-like regions of keratin A until they are denatured by basic pH (Nishikawa et al. 1998c). NMR isotope labelling of reduced cystine residues in hair keratin is possible with ^13^C-enriched methyl iodide, showing that random coil cystines are more reactive than cystines in structured regions (Nishikawa et al. 1999). Multinuclear solid state NMR has been used to study the phase composition (rigid, amorphous and interface fractions) and aspects of the reductive/oxidative processes involving disulphide linkages. In hard α-keratin, disulfide reduction followed by alkylation trapping decreases a signal near 40 ppm, assigned to Cys β-carbon (Baias et al. 2009).

Other structural techniques such as X-ray diffraction (Yang et al. 2014) and vibrational spectroscopy (Church and Millington 1996) are also of some help in understanding the relationship between keratin molecular conformation and material properties. However, in order to advance treatment and therapeutic practices, and develop rational bio-inspired material design strategies, an atomic level resolution structural model of not only the constituent polymers, but also their interfaces and interactions, is required. Attempts to gain higher resolution insight faces several challenges: lack of long range order precludes atomic resolution often available from XRD; vibrational and NMR spectroscopy are hampered by the sheer size of the constituent macromolecules, and the lack of spectral dispersion arising from repeating amino acid sequences and conformational motifs.

NMR-active stable isotope enrichment (Kainosho et al. 2006; Saxena et al. 2012; Tugarinov et al. 2006; Verardi et al. 2012; Wagner 2010), widely used in protein NMR structure determination, however, offers at least a partial way forward. Labelling facilitates multi-dimensional NMR experiments, which disperse otherwise intractably overlapped spectra into two or more frequency dimensions. Apart from the obvious increase in spectral resolution, these methods also provide structure sensitive atomic bonding and/or distance information which in favourable cases can be used as constraints for calculating detailed atomic resolution models of moderately sized biomolecules. As some of the most useful NMR-active isotopes (^13^C and ^15^N) are low natural abundance, selective, or total, isotopic enrichment is necessary. This is usually achieved by producing target biomolecules in microorganisms which will overexpress the desired construct on isotopically enriched growth media. Unfortunately, isotope enrichment of mammalian biomaterials is not yet possible this way. However, we have recently produced highly and generally enriched murine tissue and biomaterials in reasonable time and at acceptable cost (Wong et al. 2015). Briefly, the method relies on feeding a pregnant mouse on commercially available uniformly ^13^C-, ^15^N-labelled algal hydrolysate (Celtone, Cambridge Isotope Labs) supplemented with standard laboratory animal diet (fish protein hydrolysate, starch and trace elements and vitamins). At the time of weaning of the pups, all animals are culled and tissue harvested. This procedure produces high levels of protein isotope enrichment, and is particularly advantageous for labelling slower turnover tissues such as fur, quite adequate for 2D NMR. In bone, these experiments produce data which reflect chemical composition and protein structural diversity in unprecedented detail (Chow et al. 2014). Fur, also proves a highly tractable biomaterial for the application of ssNMR structure-sensitive techniques.

CP-MAS ^13^C NMR spectra of labelled fur (Wong et al. 2015) resemble those of similar keratinous materials such as equine hair (Kricheldorf and Muller 1984) and ovine wool (Yoshimizu and Ando 1990). A spectrum of labelled fur is shown in online supplementary data Fig. S1 superimposed on the largely similar spectrum of unlabelled fur from the same mouse strain. With labelling a ^15^N CP-MAS spectrum (online supplementary information Fig. S2) was obtained. However at 9.4 T there is insufficient spectral dispersion to aid structural analysis.

**Fig. 1:**
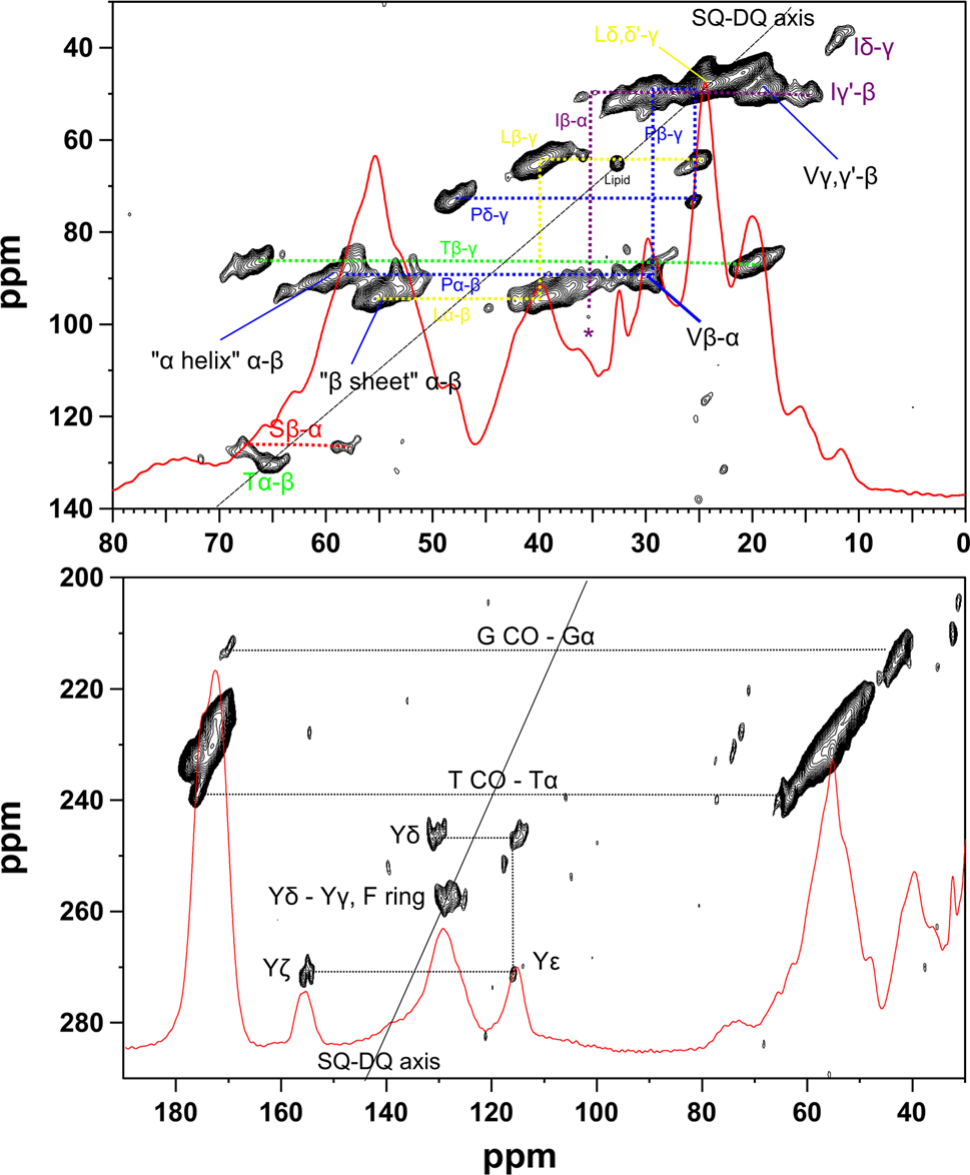
Double quantum–single quantum correlation data from labelled mouse fur. *Bottom*—One bond correlations involving amide, aromatic, and α-carbons; *Top*—One bond correlations among aliphatic carbons (the Ile Cα−Cβ cross peak is clear at higher contour levels as plotted in Fig. S3, and its position is *asterisked* in Fig. 1). Corresponding spectral regions from the 1D CP spectrum are overlaid in *red*. Fur was shaved from a mouse pup, and tightly packed directly into a 4 mm zirconia MAS rotor. Spectra showed insignificant interindividual variation. SsNMR was performed on a Bruker Avance I NMR spectrometer in a 9.4 T superconducting magnet, at 400 MHz ^1^H, 100 MHz ^13^C, MAS rate 10 kHz, ^1^H π/2 pulse 2.5 μs, contact time 2.5 ms, spin lock field 70 kHz with ramped pulse on ^1^H, spinal64 ^1^H decoupling (100 kHz RF field) during signal acquisition, chemical shifts relative to external glycine methylene at 43.1 ppm relative to TSP at 0 ppm. Double Quantum Filtering (DQF): Initial ^13^C CP as above, followed by a 70 kHz POST-C7 sequence (Hohwy et al. 1998) applied on ^13^C to excite double quantum coherence in 0.4 ms, and returned to zero quantum by another 0.4 ms POST-C7 sequence with 100 kHz Lee-Goldberg decoupling on ^1^H, and 100 kHz spinal64 decoupling during acquisition. 256 Scans were accumulated per increment, 120 increments were used, and total experiment time was about 17 h

Figure 1 depicts results from a 2D double quantum—single quantum ^13^C–^13^C correlation (DQ—SQ) experiment via POST-C7 (Hohwy et al. 1998). Briefly, the data identifies ^13^C pairs which are close in space, generally within the length of a single bond (Chow et al. 2014). As such, it indicates the covalently bonded spin systems of individual constituent amino acids or amino acid types. Atoms not directly bonded to other ^13^C’s, such as the Arg guanidinium ζ carbon, are not observed using this approach. Bonding between two ^13^Cs manifests as peaks in the 2D contour plots shown, at the sum, on the double quantum (vertical) axis of the chemical shifts of the two bonded atoms. Chemically equivalent, or near equivalent, atoms that happen to be mutually bonded thus give rise to a cross peak in the DQ dimension at twice the chemical shift of each equivalent atom. In this way, for instance, the chemically very similar and mutually bonded Phe ring carbons give rise to the broad signal envelope centred at ca. 130 ppm (in the 1D spectrum, and on the SQ horizontal axis), and produce an “autocorrelation” peak at the corresponding DQ coordinate of ca. 260 ppm. In contrast, the more spectrally dispersed Tyr ring carbons give rise to resolved sets of cross peaks corresponding to ε−ζ [pair of cross peaks at F1 coordinate ca. 271 ppm, and F2 coordinates ca. 116 (ε) and 155 ppm (ζ)], δ−ε (246 ppm in DQ dimension, and 116 ppm (ε) and 130 ppm (δ) in SQ dimension), while the cross peak between Tyr γ and δ overlaps the cross peaks from Phe δ, ϵ and ζ, at ca. 260 ppm (DQ) and 130 ppm (SQ). The expected cross peak between the quaternary Phe γ (the high frequency shoulder at ca. 137 ppm) and δ is most likely too broad and weak to be observed, as is the case for the expected Phe and Tyr γ−β connectivities. The much less abundant His and Trp residues are not observed with any certainty.

Partial splitting of the amide signals into two components (ca. 172.5 and 175 ppm in our samples) is ascribed to the co-existence of β-sheet/random coil, and α-helical, secondary structures respectively (Kricheldorf and Muller 1984). The 2D DQ–SQ experiment in addition establishes connections between signals from these two structural families and their linked α-carbon signals. In particular the unique chemical shifts of the α-carbons of Gly (43 ppm), Thr and possibly Ile (ca. 63 ppm), enable resolution, or partial resolution of their respective amide carbon signals. Correlations are assigned where possible in Fig. 1. In addition many correlations in the low frequency region of the DQ–SQ spectrum can be assigned to pairs of bonded atoms in specific amino acid residue types. The splitting of the amide signals on the basis of secondary structure is also reflected in the α-carbon spectral region, which splits into higher (β-sheet/random coil), and lower (α-helix), frequency structural regions respectively. An autocorrelation signal is also observed from biosynthetically labelled pairs of virtually equivalent lipid methylene carbons; this assignment is strengthened by chemical shift correspondence with the side chain methylene signal of a model solid phospholipid (Fig. S4).

2D PDSD experiments (Szeverenyi et al. 1982) further assist assignment (Fig. S5). The technique employs ^13^C–^13^C spin diffusion utilizing both ^13^C–^13^C and ^13^C–^1^H dipolar couplings to transfer magnetization between nearby carbons, which may not necessarily be directly bonded. The radius around a given ^13^C atom to which magnetization is efficiently transferred can be controlled by increasing the time for spin diffusion transfer between ^13^Cs; by progressively increasing this mixing time, longer ^13^C–^13^C proximities can be established, effectively mapping out the environment of a given atom. Thus, a shorter (20 ms) mixing time maps out single bond aromatic ring proximities, while a longer (100 ms) mixing time reveals longer range interatomic crosspeaks such as Cδ−Cζ, and ring carbon–amide carbon. The effects of mixing time are exemplified in Fig. S6.

In the same way variable mixing times map out proximities between amide- and α-carbons, and more distant side chain, atoms, and between side chain carbons, enabling more signals to be assigned to complete spin systems of particular residue types. Some are shown in Fig. S5, and summarized in Table 1. An alternative to the contour plot presentation for visualizing the PDSD data is to plot cross sections (rows) through “on-diagonal” peaks in which other “signals” represent proximities to these on-diagonal atoms; rows extracted from all the significant diagonal cross peaks are shown in supplementary online information Fig. S7.

**Table 1:** Assignments, secondary structural environment inferred (where possible) from δCα−δCβ, and amino acid composition, of murine fur in descending order of abundance (excluding cysteine/cystine, and tryptophan)

Amino acid	Assignments									2′ary Struct.	Mole %^*a*^
	C=O	Cα	Cβ	δCα−δCβ	Cγ	Cγ’	Cδ	Cϵ	Cζ		
E/Q^*b*^											13.58
G	**172**	**43**		43							12.95
S		**59**	**67**	−8						S	10.45
P		**60**	**30**	**30**	**25**		**48**			S	8.35
L	175	55	40	15	24		24			H	7.45
R		55^*b*^	25^*b*^	30	25^*b*^		40^*b*^		**156**	H	7.32
D/N^*b*^											6.33
Y	**174**	**54**	**36**	**18**	**130**		**130**	**116**	**156**	C	5.67
T	**175**	**63**	**65**	**-2**	**20**					H	5.58
V		**63**	**30**	**33**	**19**	**19**				H	5.41
A	173	51	24^*e*^	27						S	5.14
K		55^*b*^	32^*b*^	23	25^*b*^		25^*b*^	40^*b*^		S	3.68
F	**174**	**54**	**36**	**18**	**140** ^***c***^		**130**	**130**	**137**	C	2.95
I	173	**63**	**36**	**27**	**26**	**14**	**12**			S	2.93
H^*d*^											1.18
M^*d*^											1.02

Bold = High degree of certainty of assignment

*H* Helix, *S* Sheet, *C* Coil

^*a*^ Mean of 3 measurements, of which no standard deviation was greater than 0.4 % for any amino acid

^*b*^ No signals from these spin systems are uniquely resolved

^*c*^ High frequency shoulder on prominent signal at ca. 130 ppm

^*d*^ Not observed with confidence on account of low abundance

^*e*^ The Ala β-CH_3_ has been previously assigned at ca. 15 ppm on the basis of chemical shift and model peptide examples (Kricheldorf and Muller 1984; Yoshimizu and Ando 1990), and at ca. 18 ppm in solubilized SCMKB and denatured SMCKA (Nishikawa et al. 1998b). We do not observe the Cβ−Cα cross peak in the DQF spectrum which would occur at (F2, F1) co-ordinates of ca. (15–18, 65–70) if this assignment were correct. Accordingly we make the tentative assignment shown on the basis of the PDSD—see slice 1537 in Fig. S6. We have noticed (but not quantified) that isotope labelling of non-essential amino acids tends to be lower than of essential amino acids, probably due to *de novo* biosynthesis from unlabelled carbohydrates. This and the comparatively low abundance of Ala (5 %) may explain our failure to observe the expected Ala spin system with confidence

The 1D spectra of labelled fur closely resemble those of unlabelled material and as such offer little advantage in terms of overall structure determination, or the study of structural effects of treatments, apart from the predictable increase in sensitivity. The real power of the labelling approach is manifested by the 2D experiments which would not be possible without enrichment. This is illustrated by the differentiation between α-helical and β-sheet/random coil spectral regions exemplified by the double quantum amide—αC pairs in the DQF experiment. In particular the distinctive chemical shifts of certain atoms in a number of amino acid residues (e.g. Gly αC, Thr βC, Ser βC, Ile δC, Pro δC) results in correlation “signals” which are well resolved in one or both of the 2D experiments, and which potentially report on the chemical environment of each atom in the amino acid chain. The effective increase in resolution in the DQ dimension of the SQ–DQ correlation experiment is particularly useful, and resolves other residues apart from those already alluded to: Pro, Ile, Leu, and Val.

Resolving the spin systems of certain amino acids allows some inferences regarding their secondary structural environment. Taking advantage of the fact that the principal secondary structure families (helix, and sheet/coil) exert opposite effects on Cα, and Cβ, chemical shifts, the difference between them (δCα−δCβ, as shown in Table 1) can serve as a chemical shift index independent of the convention chosen for solid state NMR referencing. In Table S1 we reproduce the experimental chemical shifts (already shown in Table 1) of the structure dependent C=O, Cα and Cβ carbons, alongside published Chemical Shift Index (CSI) values (Wishart 2011) for the shifts of these atoms in helix, sheet, and coil, secondary structures. Where relevant signals from specific residue types are resolvable we have used δCα−δCβvalues to infer their predominant secondary structural environment, as shown in Table 1.

A combination of intermolecular covalent and non-covalent bonds between adjacent polymer chains maintains higher order structure in biomaterials, among which keratins provide particularly complex examples. In the α-keratins the most significant are covalent cystinyl disulfide bonds, and non-covalent interactions including ion pairing between acidic Asp and Glu, and basic Arg and Lys residues and hydrogen bonding between polar functional groups such as the phenolic hydroxyls of Tyr, and of polar residues with interstitial water. Changes in the physical properties and appearance of keratinous materials, importantly hair, with treatments, are a consequence of disruption and/or rearrangement of these interactions. Treatments, many used commercially, include cystinyl disulfide bond rupture by reducing agents (e.g. sodium thioglycolate), subsequently re-forming by oxidation (peroxides), and disrupting non-covalent networks and hydration by treatment with alkali, acid, or heat. Potentially any of these effects on molecular structure can be interrogated by NMR, with resolution greatly enhanced by the types of labelling approach described here. The resolution of the structurally important Tyr residues is particularly enhanced by the non-specific labelling we have described, assuring that changes in Tyr environment should be reported by NMR. Unfortunately in our universally labelled material signals from other structure-critical residues (e.g. Cys and Cys-S–S-Cys, Glu, Asp, Lys and Arg) overlap with too many other signals to be immediately useful markers of structural or chemical changes. However it is possible to modify the feeding protocol to limit the incorporation to specific labelled amino acids, or at least to these amino acids and their metabolic products in the case of the non-essentials.

In conclusion, general isotope labelling of keratin materials enables the application of powerful 2D NMR techniques which offer significant progress in determining the molecular structures and intermolecular interactions underlying its material properties. Furthermore the 2D approaches provide resolution enhancement which enables signals of a number of specific residue types to be observed and resolved. In the future this will enable a deeper understanding of the structural basis of industrial and cosmetic processes, and assist biomimetic design of new artificial materials.

## Electronic supplementary material

Below is the link to the electronic supplementary material.

Supplementary material 1 (DOCX 2720 kb)
