# Supplementary material for: Solid state NMR of isotope labelled murine fur: a powerful tool to study atomic level keratin structure and treatment effects
Source: J Biomol NMR. 2016 Oct 3;66(2):93–8. doi: 10.1007/s10858-016-0056-7 (PMC5095156; doi:10.1007/s10858-016-0056-7)
Supplement: Supplementary file 1 — Supplementary material 1 (DOCX 2720 kb) [file 10858_2016_56_MOESM1_ESM.docx]

**Supplementary information**

Solid state NMR of isotope labelled murine fur: A powerful tool to study atomic level keratin structure and treatment effects.

Wai Ching Veronica Wong, Aurimas Narkevicius, Wing Ying Chow, David G. Reid, Rakesh Rajan, Roger A. Brooks, Maggie Green, Melinda J. Duer

Fig. S1 ^13^C ssNMR spectra of isotopically labelled, and normal, mouse fur.

Fig. S2 ^15^N ssNMR spectrum of isotopically labelled mouse fur.

Fig. S3 High gain plot of low frequency region of 2D DQ-SQ correlation spectrum.

Fig. S4 Low frequency region of the 2D DQ-SQ correlation spectrum overlaid with spectrum of model phospholipid.

Fig. S5 PDSD data from labelled mouse fur.

Fig. S6 PDSD data at two mixing times

Fig. S7 Slices through PDSD datasets

Table S1 Inferring predominant secondary structure from experimental chemical shifts


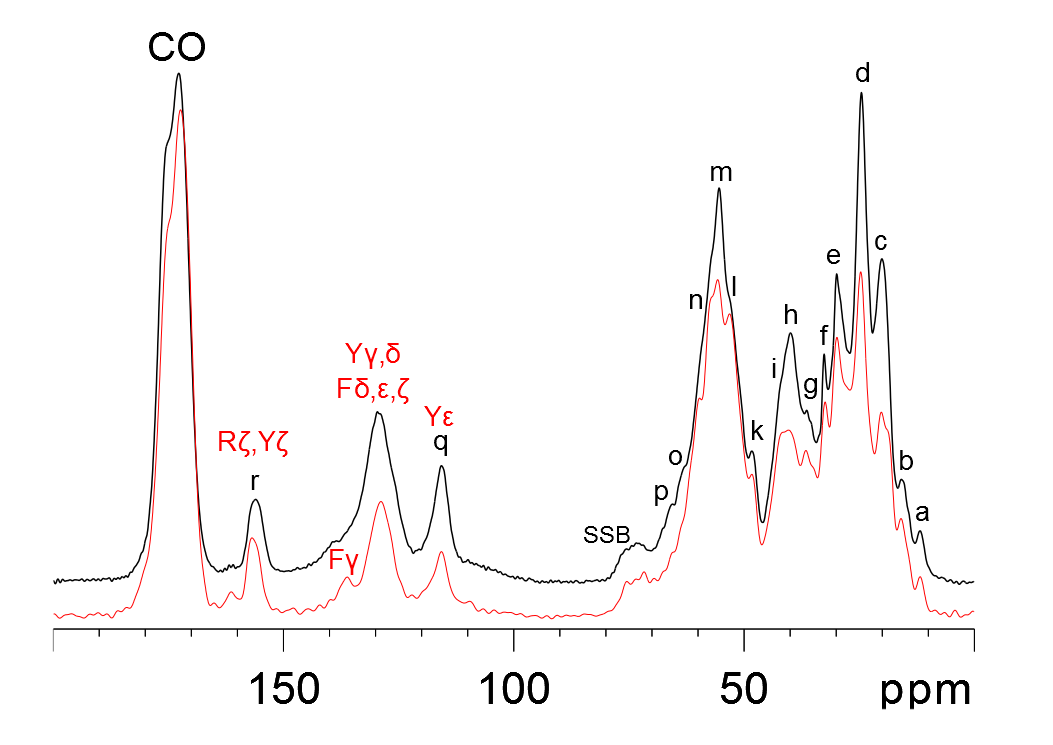


**Fig. S1** 1D ^13^C CP spectra of isotopically normal (black) and ^13^C, ^15^N labelled (red) fur (15k scans, accumulation time ca. 8½ hours). Lettering has been added to show correspondence between our data and that of Kricheldorff and Müller {Kricheldorf, 1984 #990}. Data were acquired using the CP procedure described in the legend to Fig. 1.


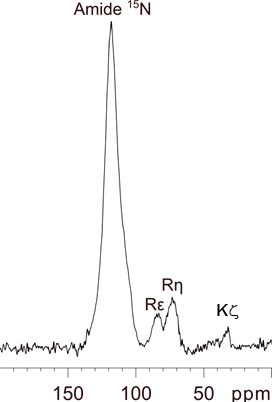


**Figure S2** ^15^N CP-MAS spectrum of ^13^C, ^15^N labelled mouse fur (40 MHz, MAS 6 kHz, contact time 5 ms, repetition time 2s, number of scans 68 k, externally referenced to glycine ^15^N signal at 32.3 ppm (relative to liquid ammonia). 64k Accumulations, acquisition time about 1½ days.


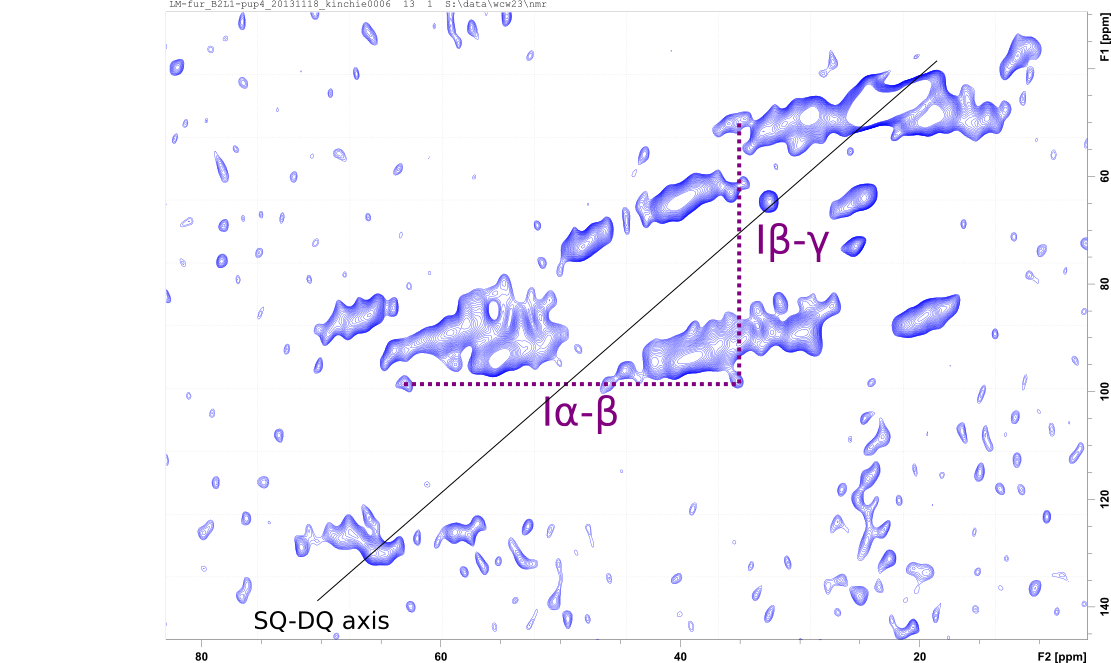


**Fig. S3** Low frequency region of the 2D DQ-SQ correlation spectrum plotted at high contour level gain to show the weak Ile Cα-Cβ correlation.


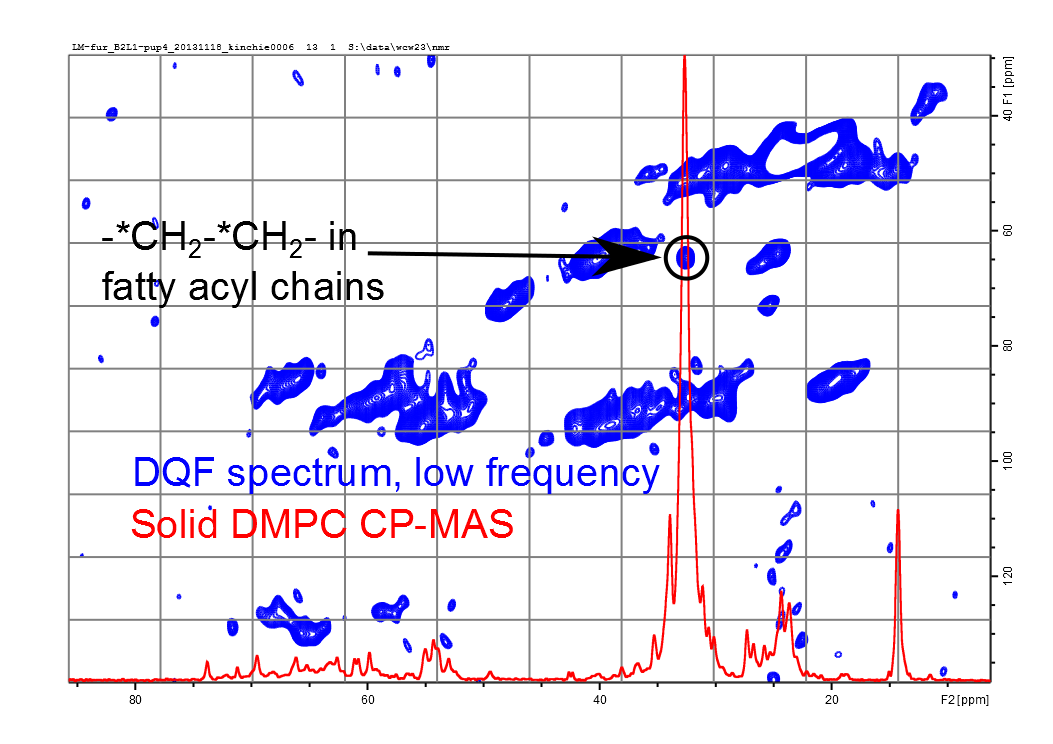


**Fig. S4** Low frequency region of the 2D DQ-SQ correlation spectrum (blue) overlaid with a 1D spectrum of solid dimyristoyl phosphatidylcholine (DMPC; Sigma, red), confirming the assignment of the methylene-methylene cross peak from lipid labelled by biosynthesis from two-carbon units generated from labelled amino acid catabolism.


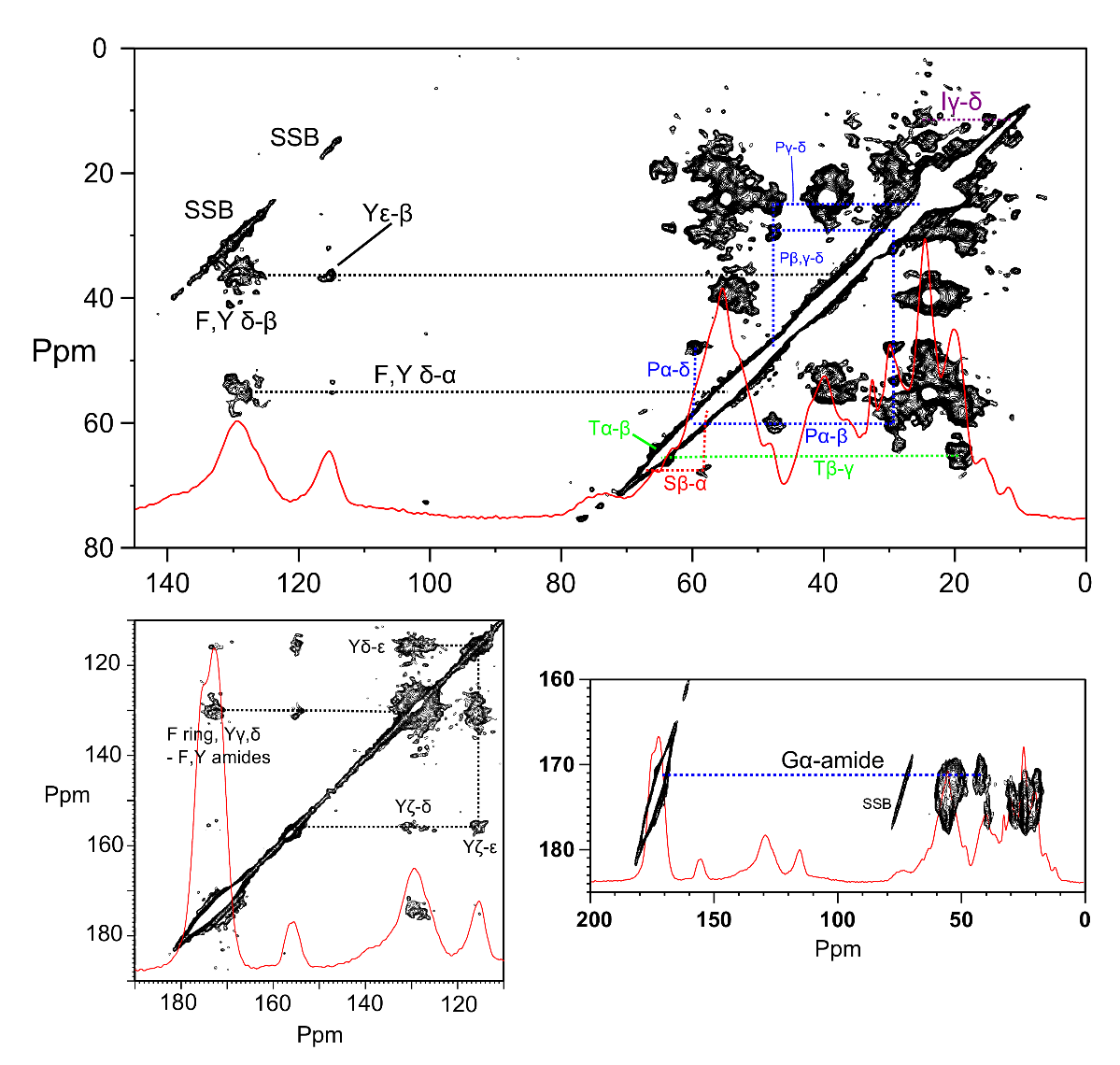


**Fig. S5** **Proton Driven Spin Diffusion (PDSD) data from labelled mouse fur.** Bottom left – Through space proximity correlations among and between aromatic and amide carbons; Bottom right – Between amide and α-carbons; Top –among and between aliphatic and aromatic carbons. Corresponding spectral regions from the 1D CP spectrum are overlaid in red. CP as for Fig. 1, followed by evolution at single-quantum coherence during the incremented delay (t­_1_), with magnetization returned to zero quantum coherence by a ^13^C 90° pulse of 3.8 μs. ^1^H decoupling was switched off during this mixing period to allow transfer of ^13^C magnetization via dipolar coupling and spin diffusion (Szeverenyi et al. 1982), with a ^13^C 90° readout pulse at the end of the mixing period. Spinal64 decoupling was applied at 100 kHz during both the incremented delay and acquisition periods. Mixing period was 100 ms, but 20 ms mixing period experiments were also recorded to interrogate the shorter carbon-carbon proximities only. Chemical shifts are relative to external glycine methylene at 43.1 ppm relative to TSP at 0 ppm. 432 Scans per increment, 360 increments, total measurement time ca. 3½ days.


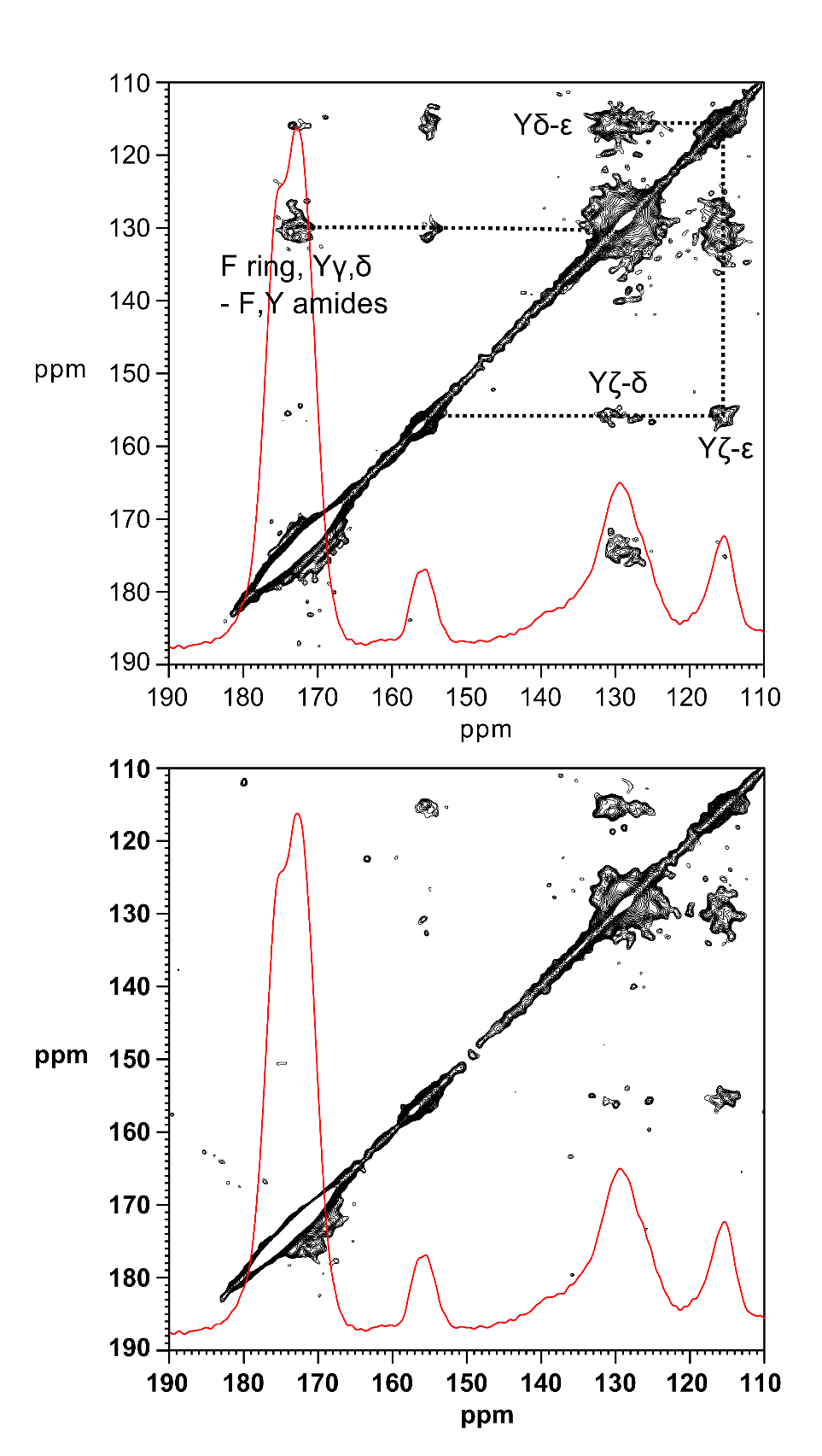


**Figure S6** A comparison of spectra acquired using short (20 ms, bottom panel, 176 scans, 360 increments measurement time ca. 1½ days) and long (100 ms, top panel, measurement time as for Fig. S5) PDSD mixing times, highlighting the response of the interaromatic, and the aromatic-amide, cross peaks. The short mixing time only produces crosspeaks that correspond to shorter interatomic distances, typically corresponding to directly bonded atoms, in contrast to the longer range connectivities revealed by the long mixing time. The 2D spectral contour plots are overlaid with the conventional 1D CP spectrum (red traces).

**Figure S7** (this and following pages). Slices from the 20 ms (blue) and 100 ms (red) mixing time PDSD datasets overlaid on a 1D spectrum (black). Spinning side band artefacts are marked as SSB or ***.

**Table S1. Inferring predominant secondary structure from experimental chemical shifts.** The Table repeats the experimental chemical shifts of the secondary structure reporter atoms C=O, Cα and Cβ, shown in Table 1 (“Assignments”), and shows the statistically derived solution state chemical shifts, as well as calculated, and experimental, δCα - δCβ values indicative of helix (H), sheet (S) and coil (C) secondary structures (see Table 7 in Wishart, D. S. Prog. NMR Spectroscopy 58, 62-87, 2011)

| **Amino Acid** | **Exptl. Shifts (Ppm)** | | | **Exptl.**  **δCα-δCβ** | **CSI δCα-δCβ*^a^*** | | | **2’ary**  **Struct** | **δ(C=O)*^b,c^*** | | | **δCα*^b^*** | | | **δCβ*^b^*** | | |
| --- | --- | --- | --- | --- | --- | --- | --- | --- | --- | --- | --- | --- | --- | --- | --- | --- | --- |
|  | **C=O** | **Cα** | **Cβ** |  | **Helix** | **Sheet** | **Coil** |  | **Helix** | **Sheet** | **Coil** | **Helix** | **Sheet** | **Coil** | **Helix** | **Sheet** | **Coil** |
| Glu E |  |  |  |  | 29.7 | 23.5 | 26.7 |  | 178.6 | 175.4 | 176.4 | 59.1 | 55.5 | 56.9 | 29.4 | 32.0 | 30.2 |
| Gln Q |  |  |  |  | 30 | 23.5 | 27 |  | 178.0 | 174.9 | 175.9 | 58.5 | 54.8 | 56.1 | 28.5 | 31.3 | 29.1 |
| Gly G | 172*^d^* | 43 |  | 43 | 46.9*^c^* | 45.2 *^c^* | 45.5 *^c^* |  | 175.5 | 172.6 | 173.9 | 46.9 | 45.2 | 45.5 |  |  |  |
| Ser S |  | 59 | 67 | -8 | -2.2 | -7.7 | -5.6 | S | 175.9 | 173.6 | 174.5 | 60.9 | 57.5 | 58.4 | 63.1 | 65.2 | 64.0 |
| Pro P |  | 60 | 30 | 30 | 34 | 30.3 | 31.6 | S | 178.3 | 176.2 | 176.9 | 65.5 | 62.6 | 63.5 | 31.5 | 32.3 | 31.9 |
| Leu L | 175*^d^* | 55 | 40 | 15 | 15.9 | 10.3 | 12.5 | H | 178.5 | 175.7 | 176.9 | 57.5 | 54.1 | 54.9 | 41.6 | 43.8 | 42.4 |
| Arg R |  | 55 | 25 | 30 | 28.8 | 22.8 | 25.7 | H | 178.3 | 175.1 | 176.0 | 58.9 | 55.1 | 56.4 | 30.1 | 32.3 | 30.7 |
| Asp D |  |  |  |  | 16.2 | 11.6 | 13.3 |  | 178.0 | 175.5 | 176.3 | 56.7 | 53.9 | 54.2 | 40.5 | 42.3 | 40.9 |
| Asn N |  |  |  |  | 16.9 | 12.6 | 14.6 |  | 176.9 | 174.6 | 175.1 | 55.5 | 52.7 | 53.2 | 38.6 | 40.1 | 38.6 |
| Tyr Y | 174*^d^* | 54 | 36 | 18 | 22.7 | 15.8 | 19 | C | 177.4 | 174.5 | 175.4 | 61.0 | 56.8 | 58.0 | 38.3 | 41.0 | 39.0 |
| Thr T | 175*^d^* | 63 | 65 | -2 | -3.3 | -9.7 | -8.5 | H | 175.9 | 173.7 | 174.7 | 65.6 | 61.1 | 61.6 | 68.9 | 70.8 | 70.1 |
| Val V |  | 63 | 30 | 33 | 34.7 | 26.9 | 29.4 | H | 177.7 | 174.8 | 175.7 | 66.2 | 60.8 | 62.1 | 31.5 | 33.9 | 32.7 |
| Ala A | 173*^d^* | 51 | 24 | 27 | 36.5 | 30.4 | 33.7 | S | 179.4 | 176.1 | 177.7 | 54.8 | 51.5 | 52.8 | 18.3 | 21.1 | 19.1 |
| Lys K |  | 55 | 32 | 22 | 26.6 | 20.8 | 23.8 | S | 178.4 | 175.3 | 176.3 | 58.9 | 55.4 | 56.6 | 32.3 | 34.6 | 32.8 |
| Phe F | 174*^d^* | 54 | 36 | 18 | 22 | 15.2 | 18.5 | C | 177.1 | 174.3 | 175.6 | 60.8 | 56.7 | 58.0 | 38.8 | 41.5 | 39.5 |
| Ile I | 173*^d^* | 63 | 36 | 17 | 27 | 20.2 | 22.3 | S | 177.7 | 174.9 | 175.6 | 64.6 | 60.1 | 61.0 | 37.6 | 39.9 | 38.7 |
| His H |  |  |  |  | 29.5 | 23.2 | 25.9 |  | 177.0 | 174.2 | 174.8 | 59.0 | 55.1 | 55.9 | 29.5 | 31.9 | 30.0 |
| Met M |  |  |  |  | 25.8 | 19.5 | 22.3 |  | 178.0 | 174.8 | 175.4 | 58.1 | 54.6 | 55.7 | 32.3 | 35.1 | 33.4 |
| Trp W |  |  |  |  | 30.7 | 24.9 | 28.1 |  | 178.1 | 175.4 | 176.2 | 60.0 | 56.4 | 57.8 | 29.3 | 31.5 | 29.7 |
| Cys C (ox.) |  |  |  |  |  |  |  |  | 176.2 | 173.6 | 174.9 | 58.0 | 55.0 | 55.6 | 39.4 | 43.9 | 41 |
| Cys C (red.) |  |  |  |  |  |  |  |  | 176.2 | 173.6 | 174.9 | 61.3 | 56.9 | 57.5 | 27.8 | 30.2 | 29.4 |

*a* – Calculated from the standard CSI shifts shown in the right hand six columns.

*b* - Statistically derived chemical shifts (solution state) for helix, sheet and coil secondary structures.

c – These solution state CSI values are dependent on the chemical shift referencing adopted and may not be rigorously compatible with experimental solid state NMR shifts.

*d* – Absolute values dependent on chemical shift referencing convention hence may not be rigorously compatible with experimental solid state NMR shifts.
